# Near‐Infrared Colloidal Quantum Dots for Efficient and Durable Photoelectrochemical Solar‐Driven Hydrogen Production

**DOI:** 10.1002/advs.201500345

**Published:** 2016-02-08

**Authors:** Lei Jin, Bandar AlOtaibi, Daniele Benetti, Shun Li, Haiguang Zhao, Zetian Mi, Alberto Vomiero, Federico Rosei

**Affiliations:** ^1^ Centre for EnergyMaterials and TelecommunicationsInstitut National de la Recherche Scientifique1650 Boul. Lionel‐BouletVarennesQC J3X 1S2Canada; ^2^ Department of Electrical and Computer EngineeringMcGill University3480 Univ. Str. WMontrealQC H3A 0E9Canada; ^3^ CNR INO SENSOR LabVia Branze 4525123BresciaItaly; ^4^ Department of Engineering Sciences and MathematicsLuleå University of Technology971 98LuleåSweden; ^5^ CSACSMcGill University801 Sherbrooke Str. W.MontrealQCH3A 0B8Canada

**Keywords:** core@shell quantum dots, hydrogen generation, near‐infrared, water‐splitting

## Abstract

**A new hybrid photoelectrochemical photoanode** is developed to generate H_2_ from water. The anode is composed of a TiO_2_ mesoporous frame functionalized by colloidal core@shell quantum dots (QDs) followed by CdS and ZnS capping layers. Saturated photocurrent density as high as 11.2 mA cm^−2^ in a solar‐cell‐driven photoelectrochemical system using near‐infrared QDs is obtained.

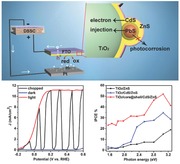

Solar energy offers a huge potential for global supply of clean and sustainable energy, reducing our dependence on fossil fuels and decreasing carbon dioxide (CO_2_) emissions.^1^, ^2^, ^3^, ^4^, ^5^, ^6^, ^7^ Photoelectrochemical (PEC) solar‐driven hydrogen (H_2_) production, which converts solar energy into H_2_ using semiconductors as active materials, is considered as a promising route, because H_2_ is a solar fuel, which combines the advantages of high energy storage densities, ease of transportation, cost‐effectiveness,^8^ and generating water as the only byproduct of H_2_ use.^9^ PEC cells perform redox reactions driven by electron–hole pairs created by incident photons, namely, the holes oxidize water/hole scavengers at the surface of the photoanode, and the electrons migrate to the counter electrode to reduce water and produce hydrogen.^10^ The ideal PEC cell is composed of inexpensive semiconducting materials with proper electronic band structure, leading to strong sunlight absorption, effective charge separation, and high photochemical stability.

TiO_2_ and ZnO are promising photocatalysts for H_2_ production by water splitting due to their appropriate energy band position and stability,^11^ yet their photoactivation requires ultraviolet (UV) light due to their wide band gap (≈3.2 eV).^12^ To improve their photocatalytic activity, chalcogenide quantum dots (QDs) optically active in the visible (Vis) range have been developed to sensitize TiO_2_, forming a heterostructured platform for PEC H_2_ generation. These QDs could expand the absorption of solar radiation into the Vis part of the solar spectrum and also provide fast exciton dissociation and charge injection to the wide band gap semiconductor due to their proper band alignment.^13^ To maximize light absorption in the broadest spectral region, lead chalcogenide QDs were applied, with band gap in the range 1.1–1.7 eV, providing a good matching of the solar spectrum.^14^ Near‐infrared (NIR) PbS QDs, which allow fast/efficient charge separation/injection in the whole UV, Vis, and NIR,^15^ can be easily grown inside a mesoporous TiO_2_ film via in situ deposition techniques, yielding a solar to H_2_ conversion efficiency of 1.15% in PEC systems, with a photocurrent density of 1.5 mA cm^−2^ and an H_2_ generation rate of 12.5 mL cm^−2^ d^−1^.^16^ However, PbS as a sensitizer suffers from problems of stability and high charge recombination.^17^ Recently, PbS QDs synthesized by a successive ionic layer adsorption and reaction (SILAR) approach and further coated by a CdS layer on TiO_2_ mesoporous film have been used as a photocatalyst to produce H_2_ at 60 mL cm^−2^ d^−1^ with a photocurrent density of 6 mA cm^−2^ under one‐sun simulated solar illumination.^18^ SILAR has several drawbacks, such as lack of precise control over QD size, difficult control on QD coverage, slow carrier injection into TiO_2_, and high charge recombination due to interfacial traps.^16^, ^17^, ^18^, ^19^ These issues can be addressed by using presynthesized colloidal QDs for sensitizing the mesoporous film.^20^ Size‐tunable QDs with high quantum yield (QY) and narrow size distribution can be synthesized via an organometallic approach in an organic solvent, with the introduction of surface ligands to passivate the QD surface.^21^ A well‐established approach, based on electrophoretic deposition (EPD), has been used to deposit these colloidal QDs onto mesoporous films,^22^ yet suffers from the degradation of the QD surface due to the damage of the protection ligand during EPD, which results in surface oxidation of QDs and their chemical etching. An elegant solution to mitigate the stability issues of colloidal QDs is to coat them with a robust inorganic shell, which inhibits surface oxidation and the formation of surface traps.^23^

Here, we describe a hybrid architecture based on a TiO_2_ mesoporous film, functionalized through EPD with colloidal PbS@CdS core@shell QDs, followed by further CdS capping via SILAR, targeting an efficient and stable hydrogen generation device. We tuned the band gap of the QDs in the range 0.9–1.77 eV by controlling their core size and modulating the conduction band edge to match the water reduction potential.^24^ The scheme of the PEC system is shown in **Figure** **1**a. Both the PbS@CdS QDs and CdS SILAR layers act as light absorbers. Photogenerated exciton dissociation occurs at the QD/oxide interface (Figure 1b), and electrons are injected into TiO_2_. Trap‐mediated electron transport occurs in the TiO_2_ mesoporous film, while electrons are collected at the front electrode and then transferred to the Pt counter electrode, where hydrogen generation takes place.^10^ Na_2_S and Na_2_SO_3_ hole scavengers in the electrolyte provide a shuttle for the photogenerated holes.^16^, ^18^, ^25^ A ZnS capping layer was applied to protect the photoabsorber from photo corrosion.^26^

**Figure 1:**
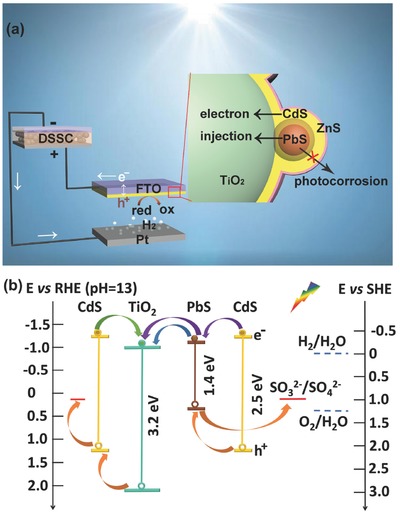
a) Scheme of the PEC device. b) Energy levels (at pH = 13) of TiO_2_,^27^ PbS QDs (3 nm in diameter),^24^, ^30^ CdS,^28^ related characteristic redox potentials.^29^ The arrows indicate the electrons and holes transfer process.

The colloidal PbS QDs were first synthesized via hot‐injection methods. Subsequently, the PbS@CdS core@shell QDs were obtained via a cation exchange approach and then dispersed in toluene, with typical QY of 40%–80%.^30^ By controlling the initial size of PbS QDs and the cation exchange parameters such as reaction temperature and time, we were able to produce core@shell QDs with the first absorption peak tunable from 0.85 to 1.54 eV (**Figure** **2**d). Based on the electron affinity of TiO_2_ (−4.1 eV), which is favorable for water reduction, and consolidated results,^21^ electrons of colloidal PbS QDs are demonstrated to be efficiently transferred into the TiO_2_ as long as the first excitonic absorption peak is above 0.9 eV (4.8 nm in diameter). This low limit for light absorption is below most *in situ* SILAR grown QDs, indicating the possibility of broadening the spectral absorbance in the proposed system.^16^, ^17^, ^18^, ^19^ Here, we used core@shell QDs with first absorption peak around 1.4 eV to demonstrate the proof of concept. The QDs have an average diameter of ≈3 nm and narrow size distribution (Figure 2a; Figure S1, Supporting Information). The blueshift of the absorption/PL peak after cation exchange is due to the shrinking of the PbS core during the replacement of Pb^2+^ by Cd^2+^ 30 The PbS core size (2.8 nm) is estimated from the position of the first excitonic peak (Figure 2b).^30^ The shell thickness (0.1 nm) is estimated from the TEM dimensions of the QDs and the calculated dimensions of the core,^31^ which is further confirmed by testing the Pb‐to‐Cd ratio with inductively coupled plasma optical emission spectrometry (ICP‐OES; see the Supporting Information). The QDs were deposited by EPD to sensitize an ≈12 μm thick TiO_2_ mesoporous layer deposited on a fluorine‐doped tin oxide (FTO) transparent conducting glass. To prevent recombination of electrons from the FTO to the oxidized species in the electrolyte, a thin TiO_2_ blocking layer was placed between the mesoporous TiO_2_ and the FTO glass.^32^ After a 2 h EPD process, a well‐separated dispersion of QDs on TiO_2_ was produced (Figure 2c), without QD aggregation, which could be detrimental for electron transfer from QDs to TiO_2_ in the PEC device.

**Figure 2:**
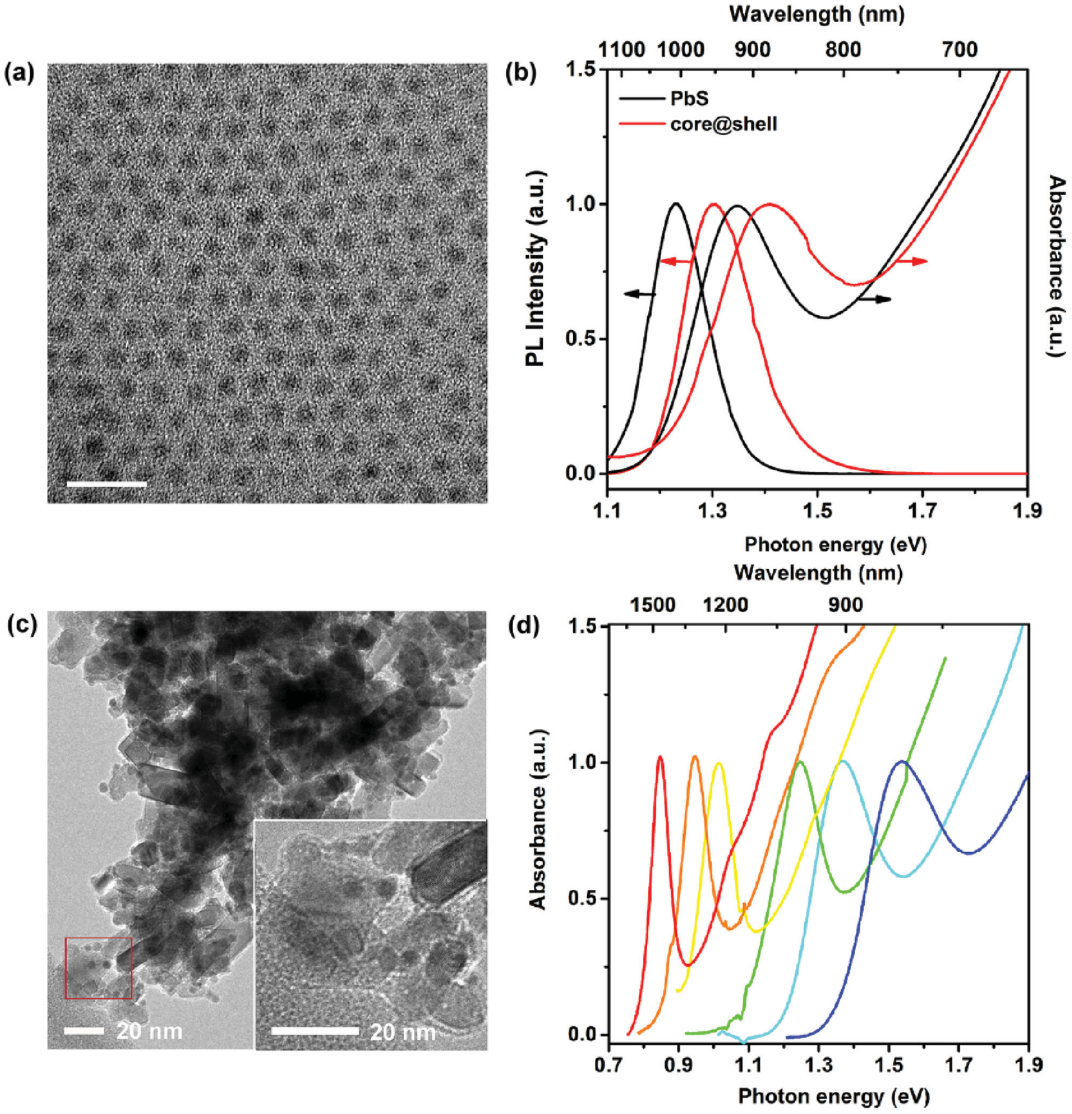
TEM images of PbS@CdS QDs a) before and c) after incorporation into TiO_2_ film by EPD; inset of (c): high‐resolution TEM image of the selected region in (c); b) The absorption and PL spectra (*λ*_ex_ = 670 nm) of pure PbS (black) and PbS@CdS QDs (red) after cation exchange. d) Representative absorption spectra of the as‐synthesized core@shell QDs with different core sizes.

The as‐prepared photoanode was further coated, after EPD, via SILAR (four cycles of CdS and two cycles of ZnS, named as TiO_2_/core@shell/CdS/ZnS), before integrating it into a PEC structure. Scanning electron microscopy (SEM) cross‐sectional imaging and related energy‐dispersive X‐ray spectroscopy (EDX) analysis (both line scan and 2D mapping) highlighted the compositional evolution of the system during its sequential formation at each step of the synthesis (Figure S2, Supporting Information). A series of benchmarking samples were also considered, namely, pure TiO_2_ mesoporous layer and TiO_2_ mesoporous layer sensitized by a CdS/ZnS SILAR film, to elucidate the role of the QDs and evaluate the contribution of the SILAR layer to the optical properties of the device. The full list of analyzed samples is reported in **Table** **1**.

**Table 1:** List of analyzed samples

Sample	Sensitizer	CdSa)	ZnSa)
TiO_2_	No one	0	0
TiO_2_/CdS	CdS film	4	0
TiO_2_/core@shell	PbS@CdS	0	0
TiO_2_/core@shell/CdS	PbS@CdS, CdS	4	0
TiO_2_/core@shell/CdS/ZnS	PbS@CdS, CdS	4	2

^a)^Number of SILAR cycles.

Before SILAR coating, the core@shell QDs after EPD show strong absorption ranging from 1.2 to 3.1 eV (**Figure** **3**b), which is very similar to its absorption spectrum in solution (Figure 2b). Further, CdS coating via SILAR (sample TiO_2_/core@shell/CdS) significantly improves the absorption of the film in the 2.25–3.1 eV range (Figure 3b). Due to the large band gap of ZnS and TiO_2_, their contribution to light absorption in the visible range is almost negligible (Figure 3b; Figure S3, Supporting Information). The PL shape and peak position almost remain unchanged after EPD compared to the solution (Figure 3b), indicating that EPD does not create any traps/surface defects affecting PL, thanks to the protection of the CdS shell. Compared to solution, the PL after QD uptake shows a strong decrease in intensity, most likely due to efficient charge transfer from QDs to TiO_2_.^21^ After further coating the core@shell QDs with the CdS (core@shell/CdS), a significant PL redshift from 1.17 to 0.92 eV (≈0.25 eV) was observed, most likely due to the creation of trap states, when the surface oleic acid ligands were modified by the presence of the inorganic CdS capping. These traps can act as alternative pathways for exciton recombination and PL generation, in addition to the standard direct band gap emission, namely, trap emission (route 1 and route 3 in Figure 3a, respectively).^33^ On the other hand, a dramatic enhancement of integrated PL intensity as high as 52‐fold was observed after CdS coating (core@shell/CdS), indicating strong inhibition of electron transfer into TiO_2_. For samples with identical optical density at the same excitation wavelength, the integrated fluorescence intensities of core@shell in TiO_2_ are reduced to about one‐fiftieth of the core@shell QDs in solution, indicating that QD‐TiO_2_ electron transfer is still the dominant process in core@shell/CdS, even if less effective, compared to core@shell before SILAR.

**Figure 3:**
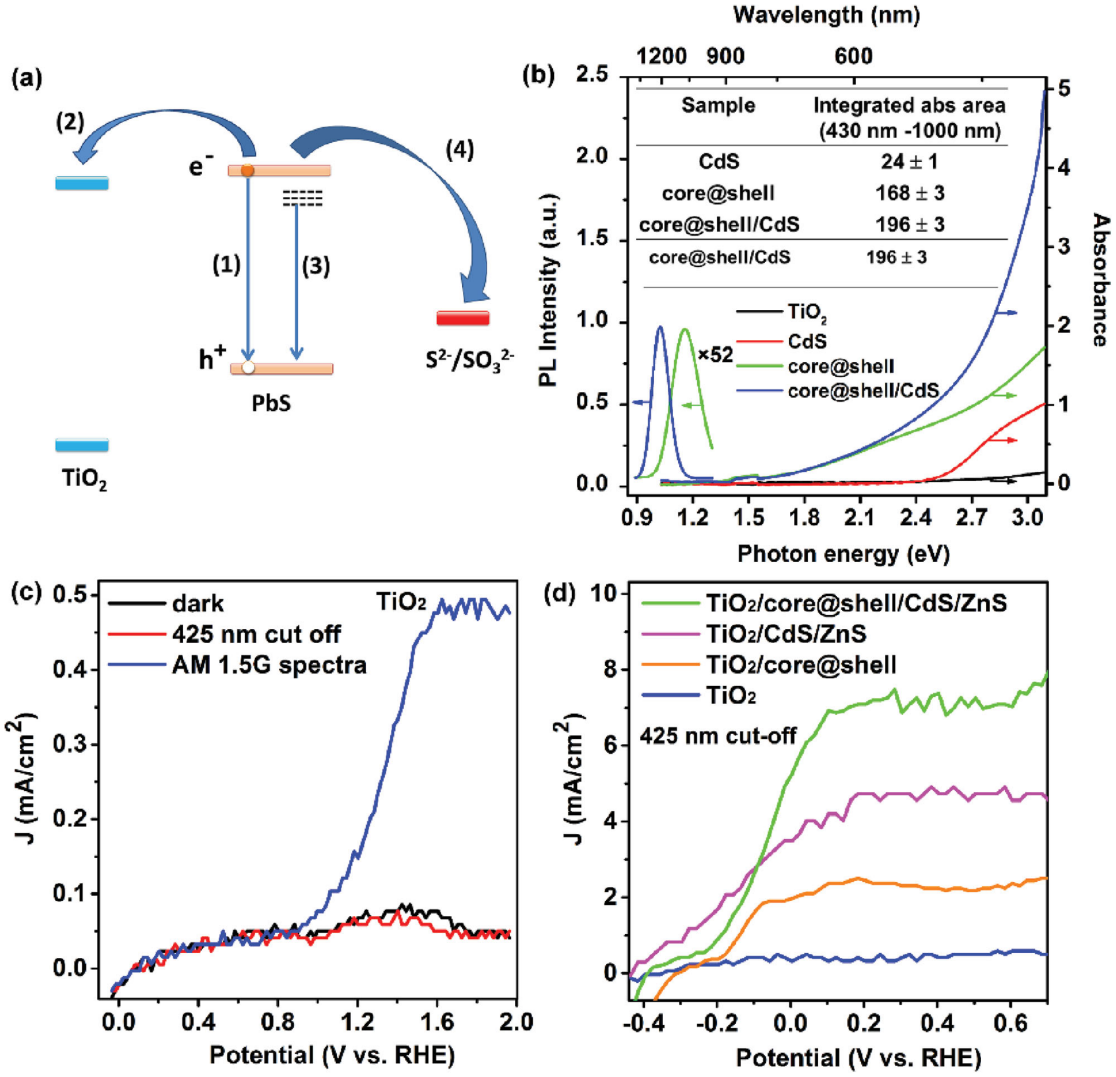
a) Photoelectron transfer pathways of PbS QDs through the band gap recombination (1); trap recombination (3); transfer to TiO_2_ (2) or recombination with electrolyte (4). b) UV–vis–NIR absorption spectra of the bare TiO_2_ film (TiO_2_) and the TiO_2_ films sensitized by CdS SILAR film (CdS), colloidal PbS@CdS QDs (TiO_2_/core@shell) and colloidal PbS@CdS QDs followed by CdS SILAR treatment (TiO_2_/core@shell/CdS). The inset table of (b) indicates the integrated absorption area for various thin films. The PL spectra of the sensitized photoanode before (TiO_2_/core@shell) and after CdS SILAR coating (TiO_2_/core@shell/CdS) are also shown in normalized form. The PL intensity of TiO_2_/core@shell was multiplied by a factor of 52 to match the one of TiO_2_/core@shell/CdS. Current density–potential dependence of the TiO_2_ film c) and the TiO_2_‐QD film photoanode d) under irradiation with long‐pass filter (*λ* > 425 nm).

The PEC behavior of the TiO_2_/core@shell/CdS/ZnS system in the dark and under illumination using a conventional three‐electrode configuration with an Ag/AgCl (saturated KCl) reference electrode and Pt counter electrode is shown in Figure 3d. An aqueous solution containing 0.25 m Na_2_S and 0.35 m Na_2_SO_3_ (pH = 13) serves as sacrificial hole scavenger. To rule out any effects of TiO_2_, a 425 nm long‐pass filter was used to measure the photoresponse under AM 1.5G illumination (100 mW cm^−2^) (Figure 3c) (the final real light intensity should be less than 100 mW cm^−2^ due to the cutoff of light below 425 nm). The photocurrent density for the bare TiO_2_ photoanode under full sun illumination without the 425 nm cutoff is 0.5 mA cm^−2^. When the filter is applied, almost no photocurrent is visible any longer, indicating that the UV radiation matching the TiO_2_ energy gap *E*_g_ is responsible for the previously observed photocurrent.

In the TiO_2_/CdS/ZnS system, a saturated photocurrent density of 4.5 mA cm^−2^ was obtained at ≈0.2 V versus reversible hydrogen electrode (RHE), comparable with a previous report for the same structure.^34^ In the TiO_2_/core@shell sample, we recorded a saturated photocurrent density of 2 mA cm^−2^, which is boosted to 7.3 mA cm^−2^ in the case of TiO_2_/core@shell/CdS/ZnS. This value is higher than the sum of the pure TiO_2_/core@shell (2 mA cm^−2^) and TiO_2_/CdS/ZnS (4.5 mA cm^−2^), suggesting a cooperative action in the composite photoanode between the NIR QDs and the overcoating layer. PbS QDs improve the visible and NIR‐light absorption (550–1000 nm), and the photogenerated electrons in the composite system can be effectively transferred to TiO_2_ nanoparticles, with an increase of more than 3.5 times in the photocurrent density. Figure 3a presents the PEC behavior of TiO_2_/core@shell/CdS/ZnS under AM 1.5G illumination without any optical filter. The positive photocurrent corresponds to hole injection from the heterostructured TiO_2_/QDs photoanode to the solution. The saturation of the photocurrent density (max 8 mA cm^−2^) occurs at ≈0.2 V versus the RHE. In this sample, a blocking layer obtained from a TiO*_x_* precursor solution was applied (see the Supporting Information). The system under high intensity (800 mW cm^−2^) simulated sun irradiation exhibits a photocurrent density of 66 mA cm^−2^ (**Figure** **4**a). We investigated the stability of our system under standard (100 mW cm^−2^) and high‐intensity (800 mW cm^−2^) light irradiation. Uncapped TiO_2_/core@shell presented a fast degradation in generated photocurrent during the first 200 s, which is highly reduced by the CdS SILAR capping layer (Figure 4b). The TiO_2_/core@shell/CdS/ZnS photoanode presents a fast transient (first 200 s irradiation) before reaching a stable photocurrent density. After 2 h light irradiation at 100 mW cm^−2^ intensity, a 30% drop in current density collected with a bias of 0.2 V versus RHE was observed (Figure 4b), ranking among the most stable QD‐based photoelectrolysis cells.^35^, ^36^, ^37^ Under high‐intensity irradiation (800 mW cm^−2^), a 13% drop in current density was detected after 10 min. A possible explanation of the reduction in photocatalytic activity could be the presence of unpassivated surface states of core@shell/CdS after ZnS treatment^38^ and/or the consumption of the sacrificial reagents (S^2−^, SO_3_^2−^).^39^, ^40^

**Figure 4:**
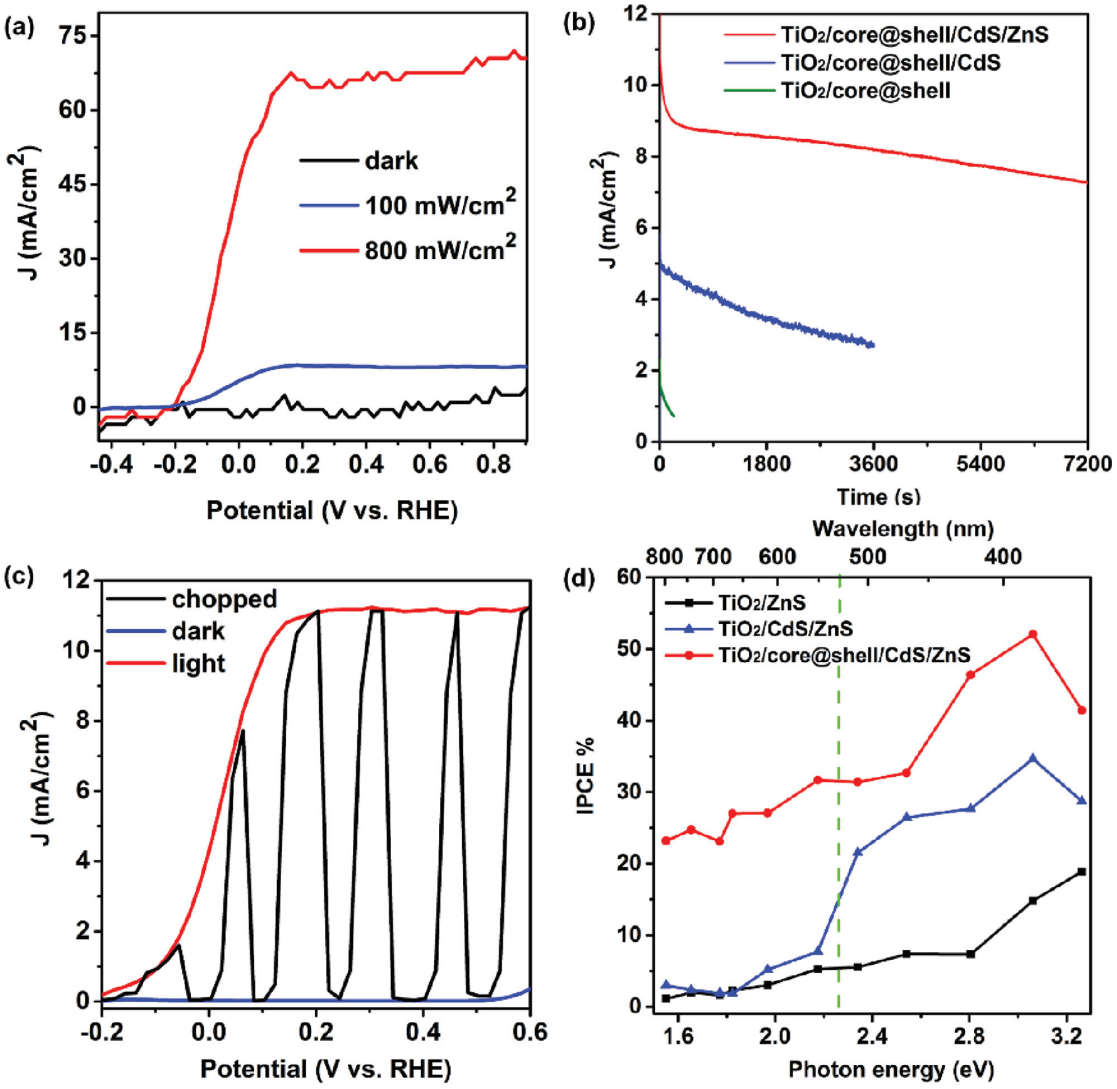
a) Photocurrent density versus the applied voltage (vs RHE) for the TiO_2_/core@shell/CdS/ZnS in the dark (black line) under AM 1.5G illumination at 100 mW cm^−2^ (blue line) as well as 800 mW cm^−2^ (red line). b) Measured current density as a function of time for the samples TiO_2_/core@shell/CdS/ZnS, TiO_2_/core@shell/CdS, TiO_2_/core@shell at 0.2 V versus RHE under 100 mW cm^−2^ illumination with AM 1.5G filter. c) *J*−*V* curves under chopped (black) and constant (red) illumination (100 mW cm^−2^). d) IPCE of the samples TiO_2_/core@shell/CdS/ZnS, TiO_2_/core@shell/CdS, and TiO_2_/core@shell at 0.2 V versus RHE as a function of the irradiation energy (wavelength). The dashed green curve highlights the low‐energy region of the solar spectrum (*λ* > 530 nm), in which light absorption and photocurrent generation are still active, thanks to the presence of the PbS@CdS core@shell QDs.

These results suggest that the CdS shell can largely enhance the photostability of the PbS core, benefiting from its isolation. Such a high stability under intense illumination offers good potential for combination with solar concentrator technology^41^ or solar thermal energy. In addition, the increasing temperature produced by solar thermal energy could decrease the necessary energy for electrolytic H_2_ production.^42^ As a consequence, the electrolysis of water could be substantially enhanced.

To optimize the PEC cell, a TiO_2_ blocking layer using commercial formulation (see the Supporting Information) was deposited between the mesoporous TiO_2_ and FTO. A saturated photocurrent density of 11.2 mA cm^−2^ was observed for 100 mW cm^−2^ light intensity (Figure 4c), most likely due to improved compactness and uniformity of the blocking underlayer. Assuming a Faradaic efficiency of unity, the corresponding hydrogen generation rate is around 112 mL cm^−2^ d^−1^, which is 86% higher than the reported PEC system using SILAR PbS/CdS QDs (60 mL cm^−2^ d^−1^) and is unprecedented for NIR QD‐based PEC for H_2_ generation.^18^ To confirm the contribution of infrared photons to the photocurrent, the incident photon to current efficiency (IPCE) was derived by current–voltage measurements (see the Supporting Information and Figure 4d). The results are consistent with the broad NIR absorption of the QDs (Figure 3b), with significant contribution to IPCE originating from the NIR region, up to 750 nm.

An external bias is still needed in our PEC system, and a substantial improvement can be expected in a stand‐alone design (Figure 1a), namely, a photovoltaic absorber unit that can be used to provide the needed bias, so that solar energy is the only source to achieve electrocatalytic H_2_ production. We applied a series of two homemade dye‐sensitized solar cells (DSSCs), providing an external open circuit voltage of ≈1.4 V (Figure S5, Supporting Information), which can be used as an external bias. No significant differences were found for the PEC behavior of the photoanode by using either the external bias supported or by using the solar cells, indicating that it is possible to make a PEC for H_2_ production using solar radiation as the only energy source.

In summary, we proposed an optimized heterostructured photoanode, to be applied in PEC cells, based on NIR‐active core@shell QDs, further capped with a CdS layer and a passivating ZnS external shell. The hybrid heterostructured photoanode can produce a photocurrent density as high as 11.2 mA cm^−2^ (equivalent to 112 mL cm^−2^ d^−1^ H_2_) by cyclic voltammetry in a three‐electrode configuration under 100 mW cm^−2^ AM 1.5G illumination, being a good candidate architecture for solar hydrogen generation. Photocurrent measurements confirmed the contribution of colloidal NIR QDs and CdS layer to H_2_ generation, extending light collection to the NIR region of the solar spectrum. Additionally, irradiation under both standard and high intensity illumination demonstrated the good stability of the system compared to other QD‐based systems in the literature, suggesting the possibility of application in solar concentrators. We further demonstrated the possibility of integrating our PEC cell in a stand‐alone device by coupling it with properly sized DSSCs to supply an external bias. Future development of water splitting by using NIR‐active QDs can focus on the optimization of charge transfer by suitable engineering of the composition/thickness of the external shell and of the surface capping agents (application of halides can be a very promising route). Other strategies to increase the efficiency of PEC systems rely on tuning core size to further broaden light absorption to the NIR region and on the removal of surface traps (for instance, by controlled annealing). Proper capping is also a key strategy to increase the long‐term stability of these systems, which is still an open issue.

## Experimental Section

A detailed description of the experimental methods is available in the Supporting Information.

*QD Synthesis*: PbS QDs with diameter ≈3.0 nm were synthesized by hot injection method by using oleic acid (OA) as ligand.^43^ PbS@CdS QDs were synthesized via a cation exchange method.^44^ The PbS@CdS was washed by ethanol and redispersed in toluene. The redispersion–precipitation procedure was repeated twice.

*TiO_2_ Film Preparation*: A thin and compact TiO_2_ layer was spin coated on FTO‐coated glass at 2000 rpm for 60 s by using the commercial solution Ti‐Nanoxide BL/SC (Solaronix) or using TiO*_x_* flat film precursor solution.^45^ Then, the films were annealed in air at 500 °C for 30 min after drying and cooled down to room temperature. A 20 nm particle size paste named as 18NR‐T (paste A, from Dyesol) and a blend of active anatase particles (≈20 nm) and larger anatase scatter particles (up to 450 nm) paste (18 NR‐AO, paste B, from Dyesol) were tape casted, forming a mesoporous film with thickness ≈12 μm, as measured by contact profilometry.

*EPD of the QDs on the TiO_2_ Film*: A pair of TiO_2_ FTO slides were vertically immersed in the QDs solution and facing each other with a distance of 1 cm. A voltage of 200 V was applied for 120 min.^22^ The samples were then rinsed several times with toluene and dried with N_2_ at room temperature. Four monolayer (ML) CdS and 2 ML ZnS were subsequently deposited using SILAR.^34^, ^46^

*Characterization*: The morphology of PbS@CdS QDs was characterized using a JEOL 2100F TEM. The composition of the films was measured on a freshly cleaved cross‐section of the TiO_2_ layers after EPD, using an Atmospheric Thin Window (ATW) EDX detector in an FEI Sirion high‐resolution SEM system operated at 10–15 kV accelerating voltage (133 eV resolution at 5.9 keV). Absorption spectra were acquired with a Cary 5000 UV–Vis–NIR spectrophotometer (Varian) with a scan speed of 600 nm min^−1^. Fluorescence spectra were taken with a Fluorolog‐3 system (Horiba Jobin Yvon) and the excitation wavelength was set at 430 nm. The Pb‐to‐Cd ratio was determined by using ICP‐OES (Perkin Elmer Model Optima 7300 DV). The PEC performance of the photoelectrodes was evaluated in a typical three‐electrode configuration, with 0.25 m Na_2_S and 0.35 m Na_2_SO_3_ as the sacrificial hole scavengers. All potentials measured with respect to Ag/AgCl were converted to the RHE scale.^18^, ^47^ The IPCE was derived from current−voltage measurements using different band‐pass optical filters (see the Supporting Information).^47^

## Supporting information

As a service to our authors and readers, this journal provides supporting information supplied by the authors. Such materials are peer reviewed and may be re‐organized for online delivery, but are not copy‐edited or typeset. Technical support issues arising from supporting information (other than missing files) should be addressed to the authors.

Supplementary
