# Supplementary material for: Near‐Infrared Colloidal Quantum Dots for Efficient and Durable Photoelectrochemical Solar‐Driven Hydrogen Production
Source: Adv Sci (Weinh). 2016 Feb 8;3(3):1500345. doi: 10.1002/advs.201500345 (PMC5021169; doi:10.1002/advs.201500345)
Supplement: Supplementary file 1 — Supplementary [file ADVS-3-1500345-s001.pdf]

Copyright WILEY-VCH Verlag GmbH & Co. KGaA, 69469 Weinheim, Germany, 2016.

Supporting Information

for *Adv. Sci.*, DOI: 10.1002/advs. 201500345

Near-Infrared Colloidal Quantum Dots for Efficient and Durable Photoelectrochemical Solar-Driven Hydrogen Production

Lei Jin, Bandar AlOtaibi, Daniele Benetti, Shun Li, Haiguang Zhao, Zetian Mi, Alberto Vomiero,* and Federico Rosei**

Supporting Information

Near Infrared Colloidal Quantum Dots for Efficient and Durable Photoelectrochemical Solar-Driven Hydrogen Production

Lei Jin[1], Bandar AlOtaibi[2], Daniele Benetti[1], Shun Li[1], Haiguang Zhao[1,3,], Zetian Mi[2], Alberto Vomiero[1,3,4,*] and Federico Rosei[1,5,*]*

1 Centre for Energy, Materials and Telecommunications, Institut National de la Recherche Scientifique, 1650 Boul. Lionel-Boulet, Varennes (QC) J3X 1S2, Canada.
2 Dept. of Electrical and Computer Eng., McGill University, 3480 Univ. Str. W, Montreal (QC) H3A 0E9, Canada.
3 CNR INO SENSOR Lab, Via Branze 45, 25123 Brescia, Italy.
4 Division of Engineering Science and Mathematics, Luleå University of Technology, 971 98 Luleå, Sweden.
5 CSACS, McGill University, 801 Sherbrooke Str. W., Montreal, QC, H3A 0B8, Canada.
*** haiguang.zhao@emt.inrs.ca, alberto.vomiero@ltu.se, federico.rosei@emt.inrs.ca**

1. Experimental:

1.1 Materials

Lead acetate trihydrate, trioctylphosphine (TOP, 90%), bis (trimethylsilyl) sulfide $(TMS)_2S$ (technical grade, 70%), cadmium oxide (99%), oleic acid (OA), 1-octadecene (ODE), and hydrochloric acid cadmium nitrate ($Cd(NO_3)_2{\times}4H_2O$, 98%), zinc nitrate hexahydrate ($Zn(NO_3)_2{\times}6H_2O$, 98%), sodium sulfide ($Na_2S{\times}9H_2O$), sodium sulfite (Na_2SO_3), methanol, toluene, ethanol, titanium isopropoxide, hydrochloric acid and isopropanol were obtained from Sigma-Aldrich Inc. Ti-Nanoxide BL/SC was bought from Solaronix. Titania paste consisting of ~20 nm nanoparticles in diameter (18 NR-T) and a blend of active anatase particles (~20 nm) and larger anatase scatter particles (up to 450 nm) paste (18 NR-AO) were supplied by Dyesol (Queanbeyan, Australia). All chemicals were used as purchased.

1.2 QD Synthesis

1.2.1 PbS QDs

PbS QDs with diameter ~3.0 nm were synthesized by hot injection method by using OA as ligand.[1] In a three-neck reaction flask, a mixture of lead acetate trihydrate (1 mmol), OA (1.2 mL), TOP (1 mL), and ODE (15 mL) were heated to 150 °C for 1 h. After the system was cooled down to ~100 °C under vacuum for 15 min, 4.8 mL of a sulphur precursor solution prepared by mixing $(TMS)_2S$ (0.5 mmol) with 0.2 mL of TOP was quickly injected into the reaction flask at 130 °C.

Subsequently, the reaction was quenched in cold water. The obtained PbS QDs were precipitated with ethanol, centrifuged to remove unreacted lead oleate and free OA molecules and then re-dispersed in toluene.

1.2.2 PbS@CdS QDs

PbS@CdS QDs were synthesized via a cation exchange method.[2] Typically, CdO (2.3 mmol), OA (2 mL) and ODE (10 mL) were heated to 255 °C under N_2 for 20 min. The clear solution was cooled down to 155 °C under vacuum for 15 min. The flask was then reopened and the N_2 flux was restored. PbS QDs suspension in toluene (1 mL, Absorbance = 3 at the first exciton peak) was diluted in 10 mL toluene, bubbled with N_2 for 30 min and then immediately heated to 100 °C. The Cd/OA mixture was added via a syringe. The solution was maintained at 100 °C for 5 minutes and then cooled down to room temperature with cold water. Then the PbS@CdS was washed by ethanol and re-dispersed in toluene. The re-dispersion–precipitation procedure was repeated two times.

1.3 TiO_2 film preparation

Fluorine doped tin oxide (FTO) coated glass substrates (Pilkington, bought from Hartford Glass Co. Inc., USA) with sheet resistance 15 Ω/square were cleaned with acetone, ethanol, thoroughly rinsed with deionized water and dried in a filtered air stream. A thin and compact TiO_2 layer was spin coated on FTO substrates at 2000 r.p.m. for 60 s by using the commercial solution Ti-Nanoxide BL/SC (Solaronix) or using TiO_x flat film precursor solution,[3] which consists of 0.23 M titanium isopropoxide (Sigma-Aldrich, 99.999%) and 0.013 M HCl solution in isopropanol (Sigma-Aldrich, >99.9%). Then the films were annealed in air at 500 ℃ for 30 min after drying and cooled down to room temperature. Then a 20 nm particle size paste, which was supplied by Dyesol (Queanbeyan, Australia) under the commercial name 18NR-T (paste A), was deposited on the top of FTO by tape casting and dried in the air for 10 min. The photoanodes were then fired on a hot plate at 120 ℃ for 10 min. A blend of active anatase particles (~20 nm) and larger anatase scatter particles (up to 450 nm) paste (18 NR-AO, paste B) was then deposited on the top of paste A, following the same procedure. The electrodes were subsequently sintered

following temperature profile at 325 °C/5 min, at 375 °C/5 min, at 450 °C/15 min and at 500 °C/30 min, forming film with thickness ~12 μm, as measured by contact profilometry.

1.4 EPD of the QDs on the TiO_2 film.

QDs were dispersed in toluene, with a pair of TiO_2 FTO slides vertically immersed in the QDs solution and facing each other. The distance between them was adjusted at 1 cm. A voltage of 200 V was applied for 120 min.[4] To wash off unbound QDs after the EPD process, the samples were rinsed several times with toluene and dried with N_2 at room temperature. In a typical SILAR deposition cycle,[5,6] Cd^{2+} ions were deposited from an ethanolic 0.05 M solution of $Cd(NO_3)_2 \times 4H_2O$. Similarly, a 0.1 M aqueous $Zn(NO_3)_2$ ($Zn(NO_3)_2 \times 6H_2O$, 98%, Sigma-Aldrich) was used as Zn^{2+} source. The sulfide sources were 0.05 and 0.1 M solutions of $Na_2S\times9\ H_2O$ in methanol/water (50/50 V/V) for Cd^{2+} ions and in DI water for Zn^{2+} ions, respectively. A single SILAR cycle consisted of 5 min of dip-coating the TiO_2 working electrode into the metal precursors (Cd^{2+} or Zn^{2+}) and subsequently into the sulfide solutions. After each bath, the photoanode was thoroughly rinsed by immersion in the corresponding solvent to remove the chemical residuals from the surface and then drying with a N_2 gun. The SILAR cycle was done 4 times for CdS and 2 times for ZnS for each sample.

1.5 Characterization

The morphology of PbS@CdS QDs was characterized by a JEOL 2100F TEM. Absorption spectra were acquired with a Cary 5000 UV-Vis-NIR spectrophotometer (Varian) with a scan speed of 600 nm/min. Fluorescence spectra were taken with a Fluorolog®-3 system (Horiba Jobin Yvon) and the excitation wavelength was set at 430 nm. For the core@shell QDs, the Pb-to-Cd atomic ratio was first determined by using inductively coupled plasma optical emission spectrometry (ICP-OES) (Perkin Elmer Model Optima 7300 DV). Based on this ratio and the overall diameter from TEM images, the diameter of the PbS core and the thickness of the shell were calculated, assuming that all QDs are spherical and contain a uniform shell. The composition of the films was measured on a freshly cleaved cross-section of the TiO_2 layers after EPD, using an Atmospheric Thin Window (ATW) energy dispersive X-ray spectroscopy (EDX) detector in a FEI Sirion high resolution scanning electron microscope (HRSEM) system operated at 10–15 kV accelerating voltage (133 eV resolution at 5.9 keV). The PEC performance of the photoelectrodes

was evaluated in a three-electrode configuration, consisting of a QD-TiO_2 thin film working electrode, a Pt counter electrode, and an saturated Ag/AgCl reference electrode. TiO_2 was printed on FTO and a Cu wire was used to connect FTO using silver paste with outer circuit. An insulating epoxy resin was used to cover the sample's surface except the active area to avoid any direct contact between the electrolyte and the conducting back-contact and/or the connecting wire. Then the sample is fully immersed in the electrolyte with pH=13, containing 0.25 M Na_2S and 0.35 M Na_2SO_3 as the sacrificial hole scavenger to prevent photocorrosion of the QDs. All potentials measured with respect to Ag/AgCl during the electrochemical measurements were converted to the reversible hydrogen electrode (RHE) scale with the following expression V_{RHE} = $V_{Ag/AgCl}$ + 0.197 + pH×(0.059) [7,8]. The photoresponse was measured from a 150 W Xenon lamp used as the light source with an AM 1.5 G filter. The sample is 2 cm far from the window of lamp case (~7 cm far from actual bulb). The light intensity measured by thermopile and 2 cm away from the lamp case is ~100 mW/cm^2. The working area of the electrode is 0.16 cm^2 All the current versus potential measurements were carried out at a 20 mV/s sweep rate.

The IPCE describes the ratio of photogenerated electrons collected by the electrodes over the number of incident monochromatic photons. To derive the IPCE values, we performed current−voltage measurements using different band-pass optical filters. IPCE can be calculated by using the following equation [7]:

$$\text{IPCE\%} = \frac{c \times h}{e} \frac{J(A/cm^2)}{\lambda(nm) \times P(W/cm^2)} \times 100$$

Where J is the photocurrent density, P is the incident radiation intensity at a given wavelength, λ is wavelength of the incident photon, c, h, and e are the speed of light, Planck's constant, and the elementary electric charge, respectively. To confirm the contribution of infrared photons to the photocurrent, band-pass filters (from Edmund Optics), which have wavelength center at 380 nm , 405 nm, 442 nm, 488 nm, 532 nm, 570 nm, 630 nm, 680 nm, 700 nm, 750 nm, 800 nm were applied. For each filter, the incident radiation intensity at the position of sample was measured by Newport power-meter.

2. Supporting Figures and Tables

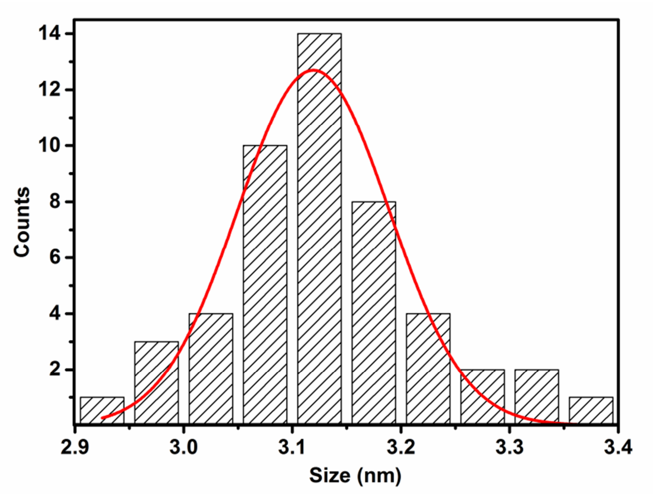

Figure S1. Size distribution of colloidal PbS@CdS QDs.

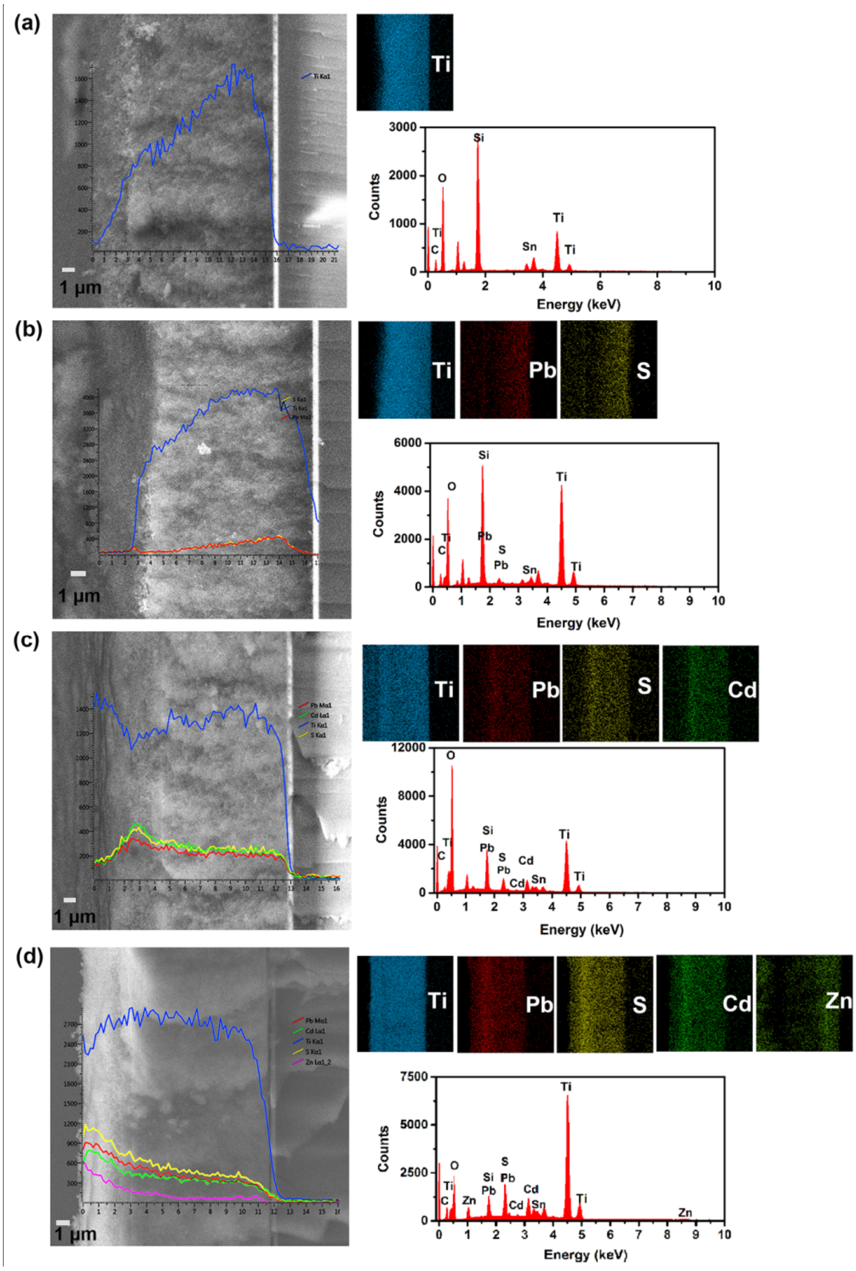

Figure S2. SEM cross-section images (left, including the EDX line scan from surface of TiO_2 to the interface between TiO_2 and FTO) and EDX analysis (right up, 2D EDX mapping and right down EDX spectrum) from (a) TiO_2, (b) TiO_2/core@shell, (c) TiO_2/core@shell/CdS, (d) TiO_2/core@shell/CdS/ZnS.

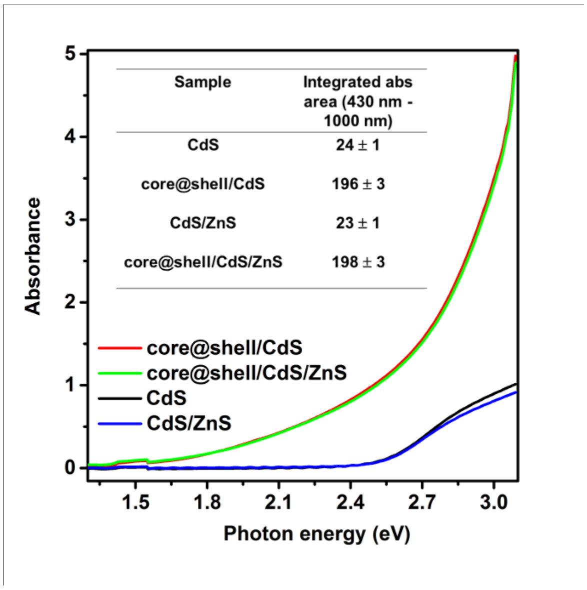

Figure S3. UV-vis absorption spectra of the TiO_2 films sensitized with CdS or CdS/ZnS SILAR treatment and co-sensitized with colloidal PbS@CdS QDs followed by CdS or CdS/ZnS SILAR treatment. Inset: integrated absorption area for various thin films.

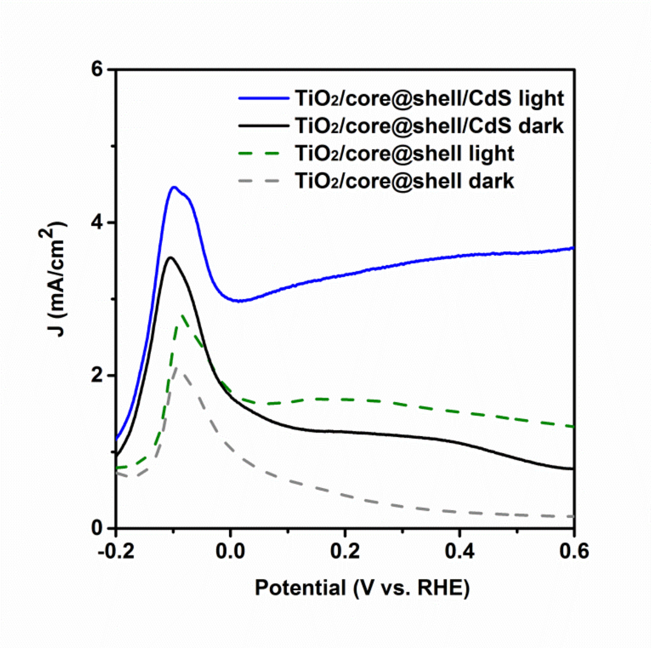

Figure S4. Photocurrent density versus the applied voltage (vs. RHE) for the TiO_2/core@shell/CdS and TiO_2/core@shell systems in the dark and under AM 1.5G illumination at 100 mW/cm^2.

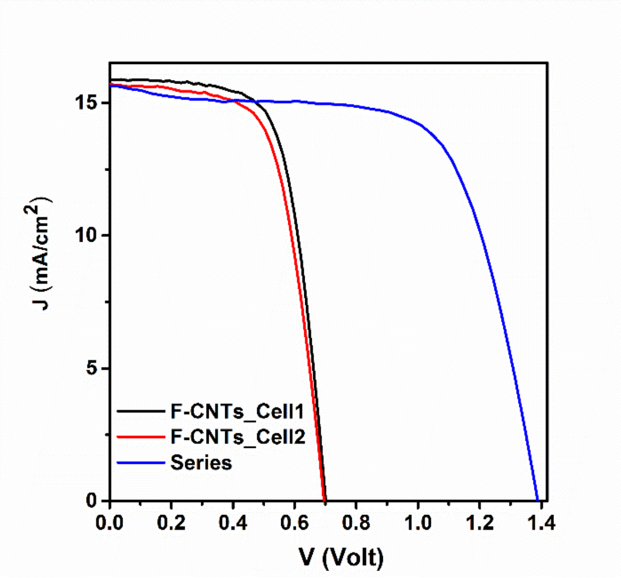

Figure S5. J-V characteristics of DSSCs with TiO_2/functionalized multi wall carbon nanotubes photoanodes under AM1.5 G illumination with light intensity of 100 mW/cm^2.

.

Table S1. Peak position, integrated area and FWHM of PL spectra in Figure 3 (b).

	With CdS	Without CdS
PL peak position (eV)	0.93 eV	1.16 eV
Integrated Area (counts)	87197	2232
FWHM (eV)	0.129	0.172

References

[1] T. Zhang, H. Zhao, D. Riabinina, M. Chaker, D. Ma, *J. Phys. Chem. C* **2010**, 114, 10153-10159.

[2] H. Zhao, M. Chaker, N. Wu, D. Ma, *J. Mater. Chem.* **2011**, 21, 8898-8904.

[3] P. Docampo, J.M. Ball, M. Darwich, G.E. Eperon and H.J. Snaith, *Nature comm.* **2013**, 4.

[4] L. Jin, H. Zhao, D. Ma, A. Vomiero, F. Rosei, *J. Mater. Chem. A* **2014**, 3, 847-856.

[5] H. Jun, M. Careem, A. Arof, *Int. J. Photoenergy* **2014**, 2014.

[6] Y.-L. Lee, C.-F. Chi, S.-Y. Liau, *Chem. Mater.* **2009**, 22, 922-927.

[7] R. van de Krol and M. Grätzel, *Photoelectrochemical hydrogen production*, Springer, **2011**, ISBN 9781461413806.

[8] R. Trevisan, P. Rodenas, V. Gonzalez-Pedro, C. Sima, R.S. Sanchez, E.M. Barea, I. Mora-Sero, F. Fabregat-Santiago, S. Gimenez, *J. Phys. Chem. Lett.* **2013**, 4, 141-146.
